# Interpretability of Clinical Decision Support Systems Based on Artificial Intelligence from Technological and Medical Perspective: A Systematic Review

**DOI:** 10.1155/2023/9919269

**Published:** 2023-02-03

**Authors:** Qian Xu, Wenzhao Xie, Bolin Liao, Chao Hu, Lu Qin, Zhengzijin Yang, Huan Xiong, Yi Lyu, Yue Zhou, Aijing Luo

**Affiliations:** ^1^The Second Xiangya Hospital of Central South University, No. 139, Renmin Road Central, Changsha, Hunan, China; ^2^School of Life Sciences, Central South University, Changsha, Hunan, China; ^3^College of Computer Science and Engineering, Jishou University, Jishou, Hunan, China; ^4^Key Laboratory of Medical Information Research, The Third Xiangya Hospital, Central South University, College of Hunan Province, Changsha, Hunan, China; ^5^Clinical Research Center for Cardiovascular Intelligent Healthcare, Changsha, Hunan, China; ^6^Big Data Institute, Central South University, Changsha 410083, China

## Abstract

**Background:**

Artificial intelligence (AI) has developed rapidly, and its application extends to clinical decision support system (CDSS) for improving healthcare quality. However, the interpretability of AI-driven CDSS poses significant challenges to widespread application.

**Objective:**

This study is a review of the knowledge-based and data-based CDSS literature regarding interpretability in health care. It highlights the relevance of interpretability for CDSS and the area for improvement from technological and medical perspectives.

**Methods:**

A systematic search was conducted on the interpretability-related literature published from 2011 to 2020 and indexed in the five databases: Web of Science, PubMed, ScienceDirect, Cochrane, and Scopus. Journal articles that focus on the interpretability of CDSS were included for analysis. Experienced researchers also participated in manually reviewing the selected articles for inclusion/exclusion and categorization.

**Results:**

Based on the inclusion and exclusion criteria, 20 articles from 16 journals were finally selected for this review. Interpretability, which means a transparent structure of the model, a clear relationship between input and output, and explainability of artificial intelligence algorithms, is essential for CDSS application in the healthcare setting. Methods for improving the interpretability of CDSS include ante-hoc methods such as fuzzy logic, decision rules, logistic regression, decision trees for knowledge-based AI, and white box models, post hoc methods such as feature importance, sensitivity analysis, visualization, and activation maximization for black box models. A number of factors, such as data type, biomarkers, human-AI interaction, needs of clinicians, and patients, can affect the interpretability of CDSS.

**Conclusions:**

The review explores the meaning of the interpretability of CDSS and summarizes the current methods for improving interpretability from technological and medical perspectives. The results contribute to the understanding of the interpretability of CDSS based on AI in health care. Future studies should focus on establishing formalism for defining interpretability, identifying the properties of interpretability, and developing an appropriate and objective metric for interpretability; in addition, the user's demand for interpretability and how to express and provide explanations are also the directions for future research.

## 1. Introduction

Clinical decision support system (CDSS), which combines clinical knowledge, patient data, and information technology, provides decision-making for health institutions to improve the quality of healthcare [1]. CDSSs are developed ranging from diagnosis [2, 3], disease management [4], and prescription [5, 6] to prediction [7–9].

Over the years, CDSSs have been attempting to integrate artificial intelligence (AI) into clinical tasks and have been recognized as the main form of application of artificial intelligence technology in the medical domain [10]. AI methodologies can be classified into two different types: knowledge-based AI and data-driven AI [11]. Some notable examples of CDSS have been developed with knowledge-based AI approaches. For example, early expert systems include MYCIN for treating bacterial infection [12], the DXplain with a large knowledge base [13], and the UpToDate based on large-scale evidence-based medicine knowledge [14]. With the application of machine learning techniques in the medical domain, data-driven AI approaches have brought tremendous advancement for CDSS during the past decades. While CDSS can be classified into different types, there are two subtypes based on the AI methodologies used: knowledge-based CDSS (KB-CDSS) and data-based CDSS (DB-CDSS) [11, 15].

KB-CDSS consists of three components: a central knowledge base, an inference engine, and end-user interface [16]. Knowledge bases are extracted from experts' knowledge represented in the forms of ontology, rules, computer interpretable guidelines (CIG), and fuzzy logic. These knowledge representations can directly express semantics and medical implications to clinicians and patients. Two common knowledge representation methods for KB-CDSS are decision rule and fuzzy logic. Wagholikar [17] developed a CDSS based on decision rules generated from guidelines to screen cervical cancer. This decision rule method provides both the traceability of the model and greater information density. Fuzzy logic handles uncertainty and imprecision by defining reality with parameters between 0 and 1 and relations between inputs and outputs [18]. The fuzzy logic method is easy to implement and understand with high accuracy and low complexity. Others also try to combine ontology and fuzzy logic to unify and normalize various types of crisp knowledge and the uncertain nature of the medical domain [19]. Generally speaking, KB-CDSS is relatively transparent and interpretable [20]. The main challenge is acquiring and upgrading the knowledge base [21].

DB-CDSS, characterized by large amounts of medical data and statistical machine learning methods, has a high level of accuracy but is less undesirable and unexplainable [22, 23]. DB-CDSSs have been used for stroke prediction [24], diabetic retinopathy grading [25], meningitis diagnosis [26], and therapeutic effects evaluation [27], and they are often described as white box or black box models [22, 28]. White-box models have the properties of linear and monotonic constraints or convey explicit information about their internal structure, such as logistic regression and decision trees [3, 22]. Based on the logistic regression model, DB-CDSSs are used for assessing patients with COVD-19 [4], diagnosing cardiovascular disease [29], and providing clinical insights of feature importance and feature correlation via coefficients. Developed based on the decision tree algorithm, online patient-oriented CRC CDSS [30] and CDSS for headache disorders [22] convey understandable information to physicians and patients. Yet, despite white box models' interpretability, its performance tends to be lower than that of black box on complex and large-scale datasets [24].

In contrast, black box models, such as nonlinear random forests, support vector machines, and neural networks, are opaque, and end-users do not know about its reasoning process and the inner connections since only the input and output can be observed during data training [3]. According to General Data Protection Regulation (GDPR), there are clauses on automated decision-making, for all individuals have the right to obtain “meaningful explanations of the logic involved” [31]. Without enabling technology capable of explaining the logic of black boxes, the DB-CDSS is hardly acceptable.

Interpretability is a crucial factor in the decision to adopt CDSS or not for healthcare institutions [32]. Clinicians and health professionals need to be assured of accuracy and safety before they can trust CDSS [33, 34]. The interpretable methods help clinicians understand the inner working mechanism of CDSS and share the results with patients in an understandable way. A survey of CDSS users reveals that interpretability significantly increases healthcare practitioners' engagement, satisfaction, and usage intentions with AI technology [35]. A variety of CDSSs using explainable AI models have been developed, such as diabetes diagnosis based on the fuzzy rule to express complex medical problems [19], heart failure survival prediction based on decision trees focusing on features importance [36], screening treatable blinding retinal diseases based on neural network by highlighting the regions of optical images [15], guideline recommendations of breast cancer based on data-driven clinical decision trees (CDTs) by transforming guidelines [37], and reviewing sleep staging results based on AI with explanations in a user-centered manner [38]. While CDSSs based on data-driven AI can often lead to more accurate results without laborious construction of the knowledge base, its insufficient interpretability presents a significant barrier to its widespread application in clinical practice [23, 39].

The explainability for AI has been a topic of concern in healthcare, and different opinions spring up from a multidisciplinary perspective [31, 40]. Some studies focus on opening the black box of medical AI [31, 41, 42]. Guidotti et al. [31] identified the different components of the family of the explanation problems, and then proposed a classification of methods of the specific explanation problem addressed, the black box model opened, the type of data used as input, and the type of explanator adopted. The proposed definition and classification of black box models should also be useful for medical AI. For interpretability in the medical field, clinical features' importance using deep Taylor decomposition for MLP, Shapley values for tree boosting, model coefficients for logistic regression [24], and activation maximization generating high-quality visualizations of classifier decisions are generally adopted for opening the black box [43]. More than just algorithm design from technological perspective, experts and clinicians have more requirements for the interpretability from medical perspective. Solutions for explainable AI include using multimodal and multicenter data fusion, expert knowledge integration, and AI to identify clinical traits [42, 44]. Kolyshkina [41] proposed a methodology CRISP-ML on the determination, measurement, and achievement of the necessary level of interpretability of ML solutions in public healthcare, taking into account public healthcare specifics, regulatory requirements, project stakeholders, project objectives, and data characteristics. To gain trustiness and acceptance of users toward medical AI, the needs of clinicians and patients for explainability get more attention. Hwang et al. [38] conducted user interviews, user observation sessions, and an iterative design process to provide clinically sound explanations in a CDSS in a user-centered design framework. It focused on what information should be contained in explanations and how explanations can be provided in the CDSS. The findings show that users concern with explanations for the input data, domain knowledge used in the task, causal information leading to output, and results influenced by input data, which should be closely related to clinical processes. Moreover, the information sources for explanations are supposed to be provided in a user-friendly and easily understandable manner, such as methods of visualization.

In addition, some systematic reviews summarize the literature of explainability for medical AI from different perspectives [32, 39, 45]. Chakrobartty and [32] provide a systematic review of the explainable AI within the medical domain focusing on methods and techniques. Moreover, more attention is being paid to issues of explainable AI from other perspectives. Amann et al. [45] adopted a multidisciplinary approach to analyze the relevance and ethical evaluation of explainability for medical AI from the technological, legal, medical, and patient perspectives to determine the need for explainability in medical AI. The finding showed that the technological perspective's explainability focused on how to attain it, whereas the legal perspective's explainability focused on informed consent, certification and approval as medical devices, and responsibility; both the physician's and patient's perspectives highlight the interaction between humans and medical AI. As the most important form of medical AI's application, the explainable AI in clinical decision support systems (CDSSs) has also raised concerns. Antoniadi et al. [39] reviewed the application of explainable AI in machine learning-based CDSS and summarized the findings of data type, preference of developers, type of explanations, and benefits of using explainable AI. These studies primarily concern on explainability of AI rather than the interpretability of CDSS, and there are still gaps regarding the relevant impacts and solutions of interpretability of CDSS. This paper intends to focus on technical solutions and medical relevant impacts of interpretability to help developers integrate explainable AI into the clinical workflow with the aim of improving the trust and acceptance toward CDSS. (1) To identify and categorize the meaning and relevant impact of interpretability of CDSS under the patient-centered principle and (2) to summarize the main interpretation methods for CDSS in clinical practice both from technological and medical perspectives.

The review is organized as follows: the Methods section outlines the search strategy, selection criteria, and quality assessment. The Results section represents the findings of our systematic review from a technological and medical perspective. The Discussion section discusses the findings, and the last section concludes the review and suggests the future direction of research.

## 2. Methods

### 2.1. Search Strategy

This literature search includes three steps: search, select, and extract. The databases for the reviewed literature include Web of Science, PubMed, Science Direct, Cochrane, Scopus, and the period ranging from 2011 to 2020. Search strategies are detailed in Table 1.

### 2.2. Selection Criteria

**Figure 1:**
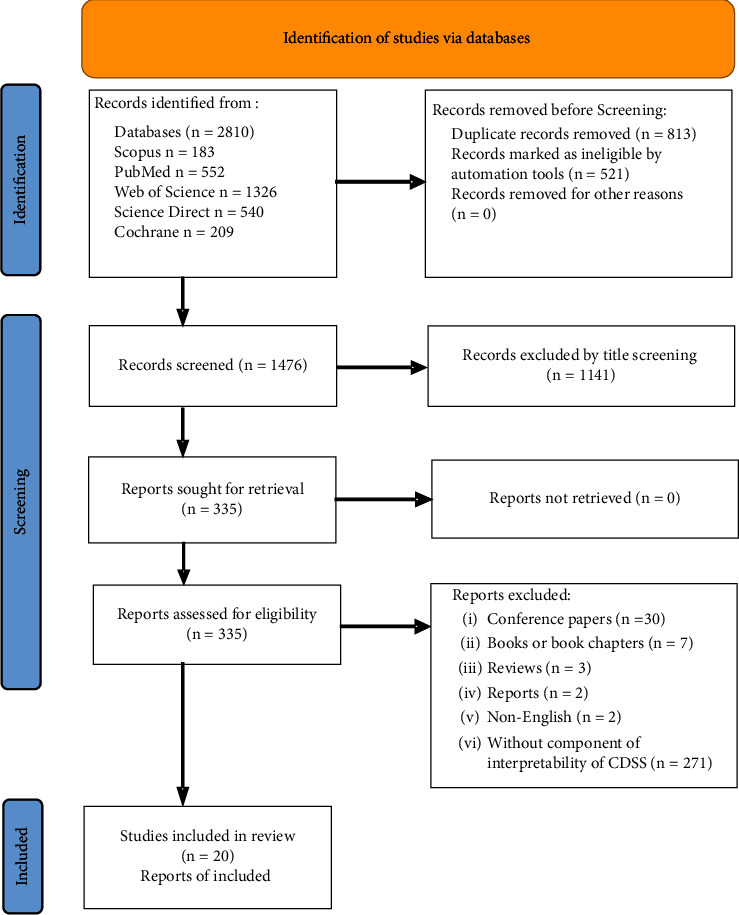
Diagram of literature search and selection processes.

Inclusion and exclusion are based on the relevance of topics, clinical tasks, evaluation, language, and types of journal articles. Specifically, articles were included if they (1) are developed for CDSS, (2) are covered by at least one of the healthcare processes (e.g. prediction, diagnosis, prognosis, risk assessment, treatment recommendations, or therapeutic management), (3) discuss the interpretability, (4) are verified and evaluated, and (5) are written in English. Studies were excluded if they (1) do not cover the application of CDSS, (2) are reviews, editorials, conference proceedings, abstracts, or book chapters, (3) lack detailed evaluation or verification, and (4) do not discuss the interpretability. In addition, two experienced reviewers screened the inclusion.  Figure 1 shows the complete processes of search and selection.

### 2.3. Data Extraction and Quality Assessment

The characteristics of included articles were identified separately by two reviewers and further verified by a senior researcher. They consist of (1) first author and publication year, (2) technological methods, (3) data sources, (4) biomarkers, (5) human-AI interaction, and (6) performance assessment. Two researchers independently assessed the quality of included articles using the widely accepted Critical Appraisal Skill Program (CASP) [46], an 11-questions tool for assessing the quality of quantitative studies [47, 48]. Multimedia Appendices 1 present the quality assessment tools used in this review. By assessing the four domains (1) objectives, (2) sample selection and methods, (3) design and results, and (4) outcomes of the research, the researchers concluded that all articles met the quality rating (the rating was >0.7) with over 80% agreement in their ratings.

## 3. Results

### 3.1. Characteristics of Included Articles

The search initially turned out 2,810 citations from the five databases. After screening, the remaining 20 articles which were published by 16 journals (e.g., “BMC medical informatics and decision-making,” “Expert systems with applications,” “International journal of medical informatics,” “Plos one,” and “IEEE Access”), were included in this review.The included articles cover a wide range of healthcare domains. Specifically, 9 articles focus on diagnosing, 4 on predicting, 2 on management, 1 on assessing health status, 2 on screening, 1 on treatment, and 1 on interpreting health examination. While these studies mainly focus on the interpretability of CDSS from the technological perspective (see Table 2), Table 3 summaries the themes related to interpretability of CDSS from a medical perspective (see Table 3).

### 3.2. Interpretability of CDSS from the Technological Perspective

All included articles discussed the interpretability of CDSS for various clinical tasks and medical scenarios. From the technological perspective, these articles examined the interpretability of CDSS along 2 themes: (1) models of CDSS based on AI and (2) interpretation method of CDSS. Table 2 summaries the themes related to interpretability of CDSS from a technological perspective.

### 3.3. Model of CDSS Based on AI

Six articles discussed interpretable knowledge-based AI methods for KB-CDSS, namely, the fuzzy logic method [19, 54], the decision rule method [17, 53, 55], and the Bayesian method [2]. Using a precise mathematical method defining reality to explicitly represent vague reality, the fuzzy logic method is an effective knowledge representation to handle the uncertainty and imprecision of medicine. The decision rule method is interpretable due to directly representing greater information density from the expert's experience and knowledge. The Bayesian model, essentially a key-value dictionary of estimated prior and conditional probabilities, is often used for inferring and ranking possible diagnoses for KB-CDSS. These three methods are transparent and interpretable, often applied for disease diagnosis. The shortcomings are also obvious: fuzzy models are difficultly partitioned and tuned out automatically without the aid of prior definition of domain experts; the decision rule-based CDSS cannot perform optimally as the inference engine totally depends on conditions matching; the Bayesian method may result in the error rate if there is an error in the prior probability and input data which determine the posterior probability of outcomes.

In contrast, the remaining 14 articles used data-driven AI methods for DB-CDSS, which can be classified as “white box model” and “black box model.” The white box models have the properties of linear and monotonic constraints, or they can reveal the inner working mechanism of the AI method. Logistic regression (LG), decision trees, and Bayes are the most often used white box models. Logistic regression methods for DB-CDSS [4, 7, 29] focus primarily on interpretability in terms of feature importance and feature correlation via coefficients. Decision trees for DB-CDSS [22, 30] represent in forms of graph structure and provide clinical interpretation of traversal rules in nodes of the tree to make decisions. The Bayesian algorithm for DB-CDSS [49] is based on prior probability for prediction. Each of these models has its advantages and disadvantages: logistic regression has a simple structure and strong interpretability for linear data and small datasets; decision trees have a transparent structure, and they can implement large-scale data sources in a relatively short time, and the Bayesian model has the advantage of stable classification efficiency for a large scale of data with fewer features.

Black box models are often referring to data-driven AI, such as support vector machine [3, 20, 52, 56], random forest [7, 8, 50], and deep learning [8, 9, 15]. Although the internal working mechanism of these models is difficult to understand, black box models can handle a huge scale of complex and interrelated data with higher performance than that of the white box model and knowledge-based AI models [3, 11]. For example, Tsao et al. [52] proposed a prediction for diabetic retinopathy based on support vector machines and artificial neural networks combined with discriminative clinical features. Kermany et al. [15] developed a predicted diagnosis with OCT image labeling based on the neural network. Recent research efforts have focused on how to open the black box to enhance the interpretability of CDSS [10, 13–15, 26].

### 3.4. Interpretation Method of CDSS

Lipton classified the interpretability of CDSS based on the AI model into two types: (1) ante-hoc methods: transparency interpretability with the aims of revealing the inner working mechanism or transparent structure of the entire model and (2) post hoc methods: interpretation for a specific decision or outcome [57]. By Lipton's classification, the ante-hoc categorized various methods with respect to the type of interpretation: decision tree [22, 30], decision rule [17, 53, 55], fuzzy inference [19, 54], Bayesian models [2], and logistic regression [4, 7, 29]; post hoc methods were divided into feature importance [8, 52], sensitivity analysis [3, 8], visualization [20, 50, 51], and activation maximization [9].

In essence, knowledge-based AI models and white box models are referred to as ante-hoc methods. Ante-hoc methods, namely, transparent boxes, directly provide local or global interpretation for CDSS leading to a safe and reliable decisions. Liu et al. [30] implemented the CRC CDSS-based decision tree algorithm, focusing on providing individualized preliminary CRC risk reports for users through a personalized interactive visualization interface. Jabez Christopher et al. [53] presented a CDSS for the diagnosis of allergic rhinitis focusing on a set of rules based on the reports of intradermal skin tests. Liu et al. [4] proposed CDSS for assessing patients of COVID-19 based on logistical regression, which provided clinical insights by means of feature importance. Müller et al. [2] proposed CDSS based on the Bayesian model for inferring and ranking possible diagnoses in terms of prior probability. Transparency consists in the level of the entire model (simulatability), at the level of individual components such as parameters (decomposability), and at the level of the training algorithm (algorithmic transparency) [31]. Ante-hoc methods, namely, transparent boxes, directly provide local or global interpretation for CDSS leading to the safe and reliable decision.

Black box models are referred to as post hoc methods. These articles categorize post hoc methods into 4 kinds of interpretations: (1) feature importance, (2) sensitivity analysis, (3) visualization techniques, and (4) activation maximization. Two of these articles examined feature importance [31]. Feature importance is a simple but effective post hoc method, as it shows the weight and magnitude of features acting as global or local interpretation in the black box [31]. Tsao et al. [52] proposed an interpretable prediction for diabetic retinopathy based on support vector machines and artificial neural networks; the model identified high-DR-risk population in terms of the discriminative feature insulin treatment and duration of diabetes selected by decision tree and logistic regression. Since feature importance would enable clinicians and patients to understand the model intuitively, approaches to investigate crucial clinical features for decision-making are highly desirable for them. In practice, the interpretable predictions for black box with varying degrees depend on the feature importance, which is drilled down and audited as the source of evidence for clinicians and patients in decision-making. However, feature importance is susceptible to noise, as well as has the disadvantage of hardly figuring out the threshold directly [58].

The second post hoc method is sensitivity analysis. Sensitivity analysis evaluates the uncertainty in the outcome of a black box with respect to the source of uncertainty inputs, and the method is generally used to develop visualization tools [59]. Esmaeili et al. [8] proposed a module based on the weight of factors analysis to provide an interpretation for predictive models, in which the sensitivity analysis focused on the information gain metric to determine the more informative features. The sensitivity analysis method is also used to determine the most important features as biomarkers for decision-making. Gaw et al. [3] employed inverse operations to identify contributing imaging features (biomarkers) in diagnosing the disease. The sensitivity analysis method focusing on the analytical pathway traces back to the contributing features and feature importance starting from the classification results. Sensitivity analysis has the advantage of the ability of finding out the most sensitive feature among the uncertain factors, coming with the disadvantage of hardly determining the true degree of the factor impact on the outcomes; in fact, the method is difficult to implement technologically, and the sensitivity analysis on AI in medicine needs further research.

The third post hoc method is visualization techniques. Visualization techniques, as representations of a specific property of the AI model, provide interpretability by revealing the inner working mechanism of black boxes [60]. Considering patients' understanding and feelings, the Dr. Answer AI for prostate cancer was developed on interpretable visualization interfaces to represent the properties of AI models and outcomes in an understandable way. In addition, the abilities of interaction on treatment plans between doctors and patients improved patients' satisfaction levels, which also built their confidence in treatment plans [50]. Tolonen et al. [51] proposed CDSS for the differential diagnosis of dementia which focused on output interpretation. The visualization tool, representing the process of decision-making, is highly desirable for end-users: clinicians. Billiet et al. [20] developed CDSS based on a colour-coded visualization, which represented the properties of assessment parameters to provide interpretable effects and interactions. In [50, 51], visualization tools represent the mechanism of decision-making. Visualization tools, by contrast, represent evaluation criteria [20]. Basically, the visualization tool is a kind of post hoc method that provides interpretability by means of showing the process of decision-making or parameters of the model. For end-user, patients and clinicians have different needs. Patients focus on information transmission and interaction with doctors in an understandable way, which will affect patients' satisfaction and confidence. In comparison, clinicians focus on understanding the mechanism of decision and the interpretation of the output of CDSS.

The fourth post hoc method is activation maximization. Activation maximization (AM) is a method used to provide interpretation for neural networks and deep neural networks. The method observes the fundamental neurons activated by input records and identifies the particular pattern of input that maximizes the activation of the certain neuron in a certain layer [61, 62]. Kaji et al. [9] developed a CDSS based on recurrent neural networks (RNNs) incorporating an attention mechanism for prediction over two weeks of patients' ICU courses. Attention maps, an activation maximization (AM) method, demonstrated when the predictor variables had the most influence on the three target variables. The predictor variables that were proxies for decision-making provided a degree of interpretability and reduced information overload for ICU physicians in a variety of important tasks. Factually, clinicians focus more on the most relevant variables for clinician decision-making and an understandable visualization tool rather than the inner structure of the neural network.

### 3.5. Interpretability of CDSS from the Medical Perspective

Interpretability is a key factor in affecting the attitudes of clinicians and patients toward CDSS based on AI [34, 63]. Four themes emerge from the reviewed articles: (1) interpretable data type, (2) biomarkers, (3) interface for human-AI interaction, and (4) needs of clinicians and patients for interpretability.

### 3.6. Interpretable Data Type

Interpretability of CDSS based on AI consists of reliable data [64], including data sources and data structure. Multiple data sources, such as hospital clinical data, online questionnaire data, scale evaluation data, patient upload data guidelines, and public dataset data, are used in the literature. Generally, hospital data are reliable, and containing high-dimensional medical information, but they are susceptible to missing values or deviations. In contrast, public datasets standardized and labeled by domain experts are of higher quality, but their availability is limited. Further research is necessary for the governance and processing of hospital data for AI applications in medicine.

Data structures used in these articles include tabular, text, images, and other formats. Tabular data can be preprocessed and calculated without a specific conversion, and the metadata associated with the tables represent medical information. Text type data are easy to read and understand by humans but difficult to compute for prediction models before they are transformed into vectors. It is necessary to use the approximate model for equivalent transformation for model interpretation. Target recognition is widely used for disease diagnosis by image-based deep learning, and the model achieves desirable performance [11, 65]. AI in medical image processing integrating with interpretation methods is an important application of CDSS in the future, which is expected to provide both interpretability and significant performance.

### 3.7. Biomarkers

Biomarkers refer to biochemical indicators of pathologic disease, pharmacologic response to treatment, or a part of a normal physiological process that can be definitively measured and assessed [66, 67]. They are important elements for clinicians and patients to understand the biological basis and to develop effective treatments [3]. Biomarker identification from medical features, by means of lasso-based feature selection [29] and inverse-transformation [3] based on linear discriminant analysis(LDA), quadratic discriminant analysis (QDA), and linear SVM (LSVM), could simplify the model and improve the diagnostic accuracy [51], as well as provide interpretability for CDSS. Biomarkers convey medical implications to clinicians and patients, helping them understand the model and promoting CDSS adoption. However, it is enormously expensive and time-consuming to discover, validate, and attain the regulatory approval of biomarkers in clinical practice. In the future, biomarker identification and validation need further research.

### 3.8. Human-AI Interaction

The interface has significant impacts on user experience, end-users' understanding, and acceptance of CDSS [68, 69]. As the operation layer of human-AI interaction, the interface has three golden rules: user's control, reduction to user's memory burden, and consistency of interface. Visualization, as a graphical interface representing the properties of AI models, helps clinicians understand the mechanism of the decision process and also provides patients with a way to get information and talk to doctors directly. CDSSs provide efficient interpretation, tailoring patients' data to their needs, and a better user experience for clinicians by using visual tools. Focusing on patients, Dr. Answer AI [50] with a user-friendly interface provided information for patients through websites and printed reports. Liu et al. [30] adopted an interactive visualization dashboard to display and interpret the risk scores and factors. It is noted that under user-centered principles for clinicians, the AI-human interfaces should be designed in an understandable way to show the processes of making decisions; also, they should be functioned with identifying errors by means of visualization of important variables. For the patient, the AI-human interface should be designed for easy accessibility of patients' information and patients' participation.

### 3.9. Needs of Clinicians and Patients for Interpretability

Clinicians and patients have various needs for interpretability in the application of CDSS. Most research focuses on issues of the black box from a technological perspective, with limited attention given to the need for interpretability from a medical perspective. In reviewing the literature, the needs of clinicians include eight categories: (1) visualization representation of a process or clinical variable proxies for clinician decision-making [2, 7, 22, 29, 30, 51, 52, 54], (2) accessibility and reliability of patients' data [4, 17, 19, 49–51, 55], (3) interface of doctors-patients or human-computer interaction for interpreting outcomes [4, 22, 50], (4) transparent structure for users to validate outputs of the model with domain knowledge [2, 7, 20], (5) identification of biomarkers for supporting decision-making [3, 29, 51], (6) feature selection distilling information overload [9, 19, 20, 52], (7) rule of representation for knowledge [2, 19, 20, 53], and (8) clinicians' needs incorporated into the clinical workflow [7]. The needs of patients for interpretability include (1) collecting patients' data of symptoms, physical exams, treatment, and reports of procedures and laboratory tests [4, 17, 19, 50], (2) interface of doctors-patients interaction for interpreting outcomes [50], (3) visualization representation of decision-making [30, 50], and (4) patient information service with informed consent [17, 50].

## 4. Discussion

### 4.1. Main Findings

There is an increasing number of studies on the explainability of various AI algorithms in healthcare. As a systematic review of the interpretability of knowledge and data-based CDSSs from technological and medical perspectives, the present study found that knowledge-based AI mainly employs fuzzy logic methods [19, 54], decision rules method [17, 53, 55], and the Bayesian method [2]. Our results indicate that the fuzzy logic method is the best fit for addressing medical uncertainty but falls short of granularities and inconsistency. The decision rule method represents knowledge intuitively in the form of “if-then” rules with the disadvantage of crude expression of record. The Bayesian model adopted the probability for inferring and ranking possible diagnoses with the disadvantage of resulting in increased error rates.

The DB-CDSS has the so-called white and black box models. The white box methods typically use logistic regression (LG) [4, 7, 29], decision trees [22, 30], and the Bayesian [49]. The logistic regression model provides clinical insights of feature importance, but it performs poorly for nonlinear datasets. The decision tree model is transparent, but it can sometimes overfit. The black box models contain the support vector machine [3, 20, 52, 56], random forest [7, 8, 50], and deep learning [8, 9, 15]. The sheer number of articles in this review (14 DB-CDSS versus 6 KB-CDSS) demonstrates that DB-CDSSs have received more attention from the researchers, signaling the potential of data-driven AI technology in health care application, even though it is handicapped by the lack of interpretability. Some researchers ventured into a hybrid model of data-driven AI and knowledge-based AI to keep trade-off performance and interpretability of CDSS in clinical practice [11], and more studies are needed in this line of research.

Interpretability is essential for the application of CDSS. Two interpretation methods of CDSS, ante-hoc methods and post hoc methods, are often used in the literature. Ante-hoc methods include decision tree [22, 30], decision rule [17, 53, 55], fuzzy inference [19, 54], Bayesian models [2], or logistic regression [4, 7, 29]. As ante-hoc methods, fuzzy logic, decision rule, and Bayesian are transparent and interpretable models. However, the performances of this kind of CDSS tend to that of the black box [3]. Post hoc methods are the interpretation method aiming to provide interpretability for the black box. These methods include feature importance [8, 52], sensitivity analysis [3, 8], and visualization [20, 50, 51]. Feature importance shows the weight and magnitude of features but is susceptible to noise. Sensitivity analysis evaluates the uncertainty in the outcome of a black box with respect to the source of uncertainty inputs but is difficult to implement technologically. Visualization, as representation of the inner working mechanism or parameters, provides interpretability for users. Activation maximization provides interpretation for neural networks and deep neural networks.

For multisources and heterogeneous structures, interpretability is comprised of hospital data with dependable and high-dimensional medical information, tables such as matrices that are simple to preprocess, and superior deep learning performance. Biomarkers, biochemical indicators of pathologic disease, pharmacologic response to treatment, or a part of the normal physiological process, convey medical implications to clinicians and patients. In fact, it is enormously expensive and time-consuming to discover, validate, and attain the regulatory approval of biomarkers in clinical practice. In the future, biomarker identification and validation need further research. Interfaces and visualization of the decision-making process or important variables have important impacts on the user experience, end-user understanding, and acceptance of CDSS.

Meeting the diverse needs of clinicians and patients for interpretability should be the goal of CDSS developers. Clinicians expect CDSS to help make decisions, identify, and avoid errors. Thus, their needs for interpretability focus on visualization representation, accessibility and reliability of patients' data, transparent structure, biomarkers, feature selection, and the rule of representation for knowledge. In contrast, for patients, CDSS should facilitate informed consent and enhance patient participation. The patients' needs for interpretability are simpler than that of clinicians, and they mainly care about patients' data, the interface of doctors-patients interaction in interpreting outcomes, visualization representation, and functioning with patient information service with informed consent.

### 4.2. Research Gaps

It is noted that there are four types of challenges and gaps associated with the clinical implementation of the interpretability of CDSS in practices. Firstly, there is no consensus on what the interpretability of CDSS is [39], and the definition of interpretability is often limited to opening the black box from the technological perspective rather than taking multidisciplinary fields into account in medical application [45]. Future research should provide a common formalism for defining interpretability and identifying the properties of interpretability. Secondly, how to evaluate and verify the interpretability of CDSS is another challenge that we can face. Existing studies focus on some subjective methods to evaluate the interpretability of AI-based CDSS, such as user experience, satisfaction, trustiness, and acceptance in the system [70]; however, the evaluation system of interpretability is still in shortage of appropriate and objective metrics. The evaluation system of interpretability requires further study. In addition, there is limited research concerning the need of users for explanations, especially focusing on what information and data should be contained in explanations. The users' concerns, such as explanations for input data, multidisciplinary knowledge used in the clinical task, casual information about output, and easily understandable interfaces, should be paid more attention. Finally, the biggest challenge for designers is how to express and provide explanations for users. Interpretation strategies should adhere to the principle of inferring step-by-step, explanation capacity, and user-familiar terms to gain user acceptance. Data-driven AI in cooperation with domain knowledge [11, 20] and interactive visualization in clinical processes [7, 51] are the two directions of research for the interpretability of AI-CDSS in the future.

### 4.3. Limitations

Despite a comprehensive approach in the literature search, the study has several limitations. First, the search query did not use MeSH terms because of the lack of consistent terminology. We may miss out some relevant studies. Secondly, only articles written in English were reviewed, leaving research in other languages out. As a result, the review might miss some important development in this field.

## 5. Conclusions

In conclusion, this review explores the meaning of the interpretability of CDSS and summarizes the current methods for improving interpretability from technological and medical perspectives. The results contribute to the understanding of the interpretability of CDSS based on AI in health care. As a core requirement, the interpretability of CDSS calls for a transparent structure of models, an understandable relationship between input and output, and enhanced explainability for AI algorithms from the technological perspective, as well as data sources, biomarkers, AI-human interaction. Furthermore, the interpretability of CDSS is influenced by the physicians' and patients' needs for it. Future studies should focus on establishing formalism for defining interpretability, identifying the properties of interpretability, and developing an appropriate and objective metric for interpretability; in addition, the user's demand for interpretability and how to express and provide explanations are also the directions for future research.

## Figures and Tables

**Table 1:** Search and filtering strategies.

Database	Search strategy	Filtered by
Web of Science	AB = (“CDSS” OR “clinical decision support system” OR “clinical decision systems” OR “CDS”) and AB = (“Interpret^∗^” OR “explain^∗^”)	AB AND year published
PubMed	(“CDSS” OR “clinical decision support system” OR “clinical decision systems” OR “CDS”) and (“interpret^∗^” OR “explain^∗^”) title/abstract	All field AND year published
ScienceDirect	(CDSS OR clinical decision support system OR clinical decision systems OR CDS) and (Interpretability OR interpretable OR explainability OR explainable)	Title, abstract, or author-specified keywords AND year published
Cochrane	(“CDSS” OR “clinical decision support system” OR “clinical decision systems” OR “CDS”) and (“Interpret^∗^” OR “explain^∗^”)	Titles, abstract, or subject AND year published
Scopus	(“CDSS” OR “clinical decision support system” OR “clinical decision systems” OR “CDS”) and (Interpretability OR interpretable OR explainability OR explainable)	TITLE-ABS-KEY AND Year published

**Table 2:** Summary of interpretability of CDSS from the technological perspective.

Ref.	CDSS based on AI	Interpretation method	CDSS type
Saak et al. [7]	Lasso regression, elastic nets random forests	Feature importance	DB-CDSS White box
Raja and Asghar [49]	Cooperative Bayesian game-theoretic	Fuzzy genetics	DB-CDSS White box
Liu et al. [4]	Multiclass logistic regression	Feature importance	DB-CDSS White box
Vandewiele et al. [22]	Decision tree, annotated with expert knowledge base	Decision tree	DB-CDSS White box
Liu et al. [30]	Decision tree	Decision tree visualization dashboard	DB-CDSS White box
McRae et al. [29]	Cardiac score card based on lasso logistic regression	Lasso logistic regression model	DB-CDSS White box
Esmaeili et al. [8]	Random forest, Naïve Bayes, K-NN, and deep learning	Sensitivity analysis	DB-CDSS
Rho et al. [50]	Random forest k-nearest neighbors logistic regression	Interface (visualization)	DB-CDSS
Kaji et al. [9]	Deep learning long short-term memory recurrent neural networks (RNNs)	Activation maximization	DB-CDSS
Kermany et al. [15]	Neural network	Feature importance	DB-CDSS
Tolonen et al. [51]	DSI classifier, RUSBoost	Visualization	DB-CDSS
Billiet et al. [20]	DSI classifier combination of complex machine learning	Interval coded scoring with toolbox interface (visualization)	DB-CDSS
Tsao et al. [52]	Decision trees, support vector machines, artificial neural networks, and logistic regressions	Features weights	DB-CDSS
Gaw et al. [3]	LDA, QDA, and SVM (LSVM)	Sensitivity analysis (biomarker identification)	DB-CDSS
Müller et al. [2]	Bayesian for inferring and ranking possible diagnoses	Bayesian visualization	KB-CDSS
El-Sappagh et al. [19]	Ontology-fuzzy rule-based	Fuzzy logic	KB-CDSS
Jabez Christopher et al. [53]	Decision rule	Decision rule	KB-CDSS
Wagholikar et al. [17]	Free-text rule base and guideline rule base	Decision rule	KB-CDSS
Esposito et al. [54]	Fuzzy logic	Fuzzy logic	KB-CDSS
Kuo and Fuh [55]	Decision rule	Decision rule	KB-CDSS

**Table 3:** Summary of interpretability of CDSS from the medical perspective.

Ref.	Data source	Data type	Biomarkers	Human-AI interaction
Saak et al. [7]	595 provided by the Hörzentrum Oldenburg GmbH (Germany).	Tabular		Visualization of the functional aspects
Raja and Asghar [49]	Wisconsin Breast Cancer, Indian Diabetes, Cleveland Heart	Tabular		
Liu et al. [4]	2243 datasets demographic information, clinical symptoms, contact history blood tests CRP, lung CT reports	Tabular		Mobile terminal apps for the patient-end and GP-end
Kaji et al. [9]	MIMIC-III Clinical Dataset, patient ICU stays (*n* = 56,841), time steps (*n* = 14), and features (*n* ≤ 225) from Beth Israel Deaconess Medical Center	Tabular		
El-Sappagh et al. [19]	60 patients distributed as 53% diabetic and 47% nondiabetic from hospitals of Mansoura University, Egypt	Tabular		
Tolonen et al. [51]	504 patients from the Amsterdam Dementia Cohort, subjective cognitive decline as controls	Tabular	Biomarker: Neuropsychological tests, CSF samples, and both automatic and visual MRI ratings	PredictND tool offering a visualization of its decision-making process
Billiet et al. [20]	Acute inflammation breast cancer cardiotocography chronic kidney disease Indian liver	Tabular		Users interacting with the training procedure by graphical toolbox
Tsao et al. [52]	536 selected patients in “DM shared care” database	Tabular		
McRae et al. [29]	579 patients with 6 risk factors and 14 biomarker measurements from AMI diagnosis in the Texas Medical Center (TMC) in Houston, TX	Tabular	Biomarkers cTnI, creatine, kinase MB, C-reactive protein, myeloperoxidase, myoglobin, BNP, adiponectin, CD40 ligand, interleukin-1 beta, matrix metalloproteinase 9, regulated on activation normal T cell, soluble intracellular adhesion molecule 1, tumor necrosis factor alpha	Panel of biomarkers expressing CVD progression
Kuo and Fuh [55]	Computer databases of Hospital Information System (HIS) and Laboratory Information System (LIS)	Tabular		Blackboard control converting the results to human readable text with familiar interface
Esmaeili et al. [8]	2441 mammography reports from Imam Khomeini Hospital	Text		
Rho et al. [50]	7,128 clinical data of prostate cancer from EMR treated with radical prostatectomy	Text		Patients obtaining report, determining treatment, predicting the outcome with a user-friendly interface
Vandewiele et al. [22]	Migbase dataset to questionnaires of 849 different patients in Turkey	Text		Physician and patients with user-friendly manner by visualizations
Jabez Christopher et al. [53]	872 patients allergic symptoms for this study	Text		
Wagholikar et al. [17]	Free-text rule base using 49293 Pap test reports in the Mayo Clinic, Rochester, EMR	Text		
Esposito et al. [54]	Interviews, questionnaires, and observations	Text		
Müller et al. [2]	2000 ICD-10 coded diseases and 450 RX-Norm coded medications, SNOMED-CT and LOINC	Terms		Diagnosis: user interface for finding best diagnosis for input symptoms
Liu et al. [30]	Colorectal Cancer Risk Assessment Tool CCRAT)			CRC risk to users by interactive visualization interface
Kermany et al. [15]	207,130 OCT images5,232 chest X-ray 4686 patients	Image		
Gaw et al. [3]	106 MRI data 57 migraine and 49 healthy controls from Mayo Clinic Arizon and Washington University	Image	Biomarker identification Area (MRI), thickness (MRI) Volume (MRI), Resting-state functional, connectivity (fMRI)	

## Data Availability

A systematic search was conducted on the interpretability-related literature published from 2011 to 2020 and indexed in the five databases: Web of Science, PubMed, ScienceDirect, Cochrane, and Scopus.

## Supplementary Materials

Supplementary Materials Multimedia Appendices 1: Critical Appraisal Skills Programme (CASP) quality assessment for qualitative studies.
