# Supplementary material for: Interpretability of Clinical Decision Support Systems Based on Artificial Intelligence from Technological and Medical Perspective: A Systematic Review
Source: J Healthc Eng. 2023 Feb 3;2023:9919269. doi: 10.1155/2023/9919269 (PMC9918364; doi:10.1155/2023/9919269)
Supplement: Supplementary Materials — Multimedia Appendices 1: Critical Appraisal Skills Programme (CASP) quality assessment for qualitative studies. [file 9919269.f1.pdf]

Multimedia Appendix 1: CASP(Critical Appraisal Skills Program) quality assessment for qualitative studies

**No**	**Question**	Saak(2020)[23]	Raja(2020)[24]	liu(2020)[25]	Esmaeili(2020)[26]	Rho(2020)[13]	Kaji(2019)[27]	Müller(2019)[28]	van de wiele(2018)[29]	Liu(2018)[30]	El-Sapagh(2018)[31]	Toloonen(2018)[32]	Billiet(2018)[33]	Kermany(2018)[34]	Tsao(2017)[35]	Gaw(2017)[36]	McRae(2016)[37]	Jabez(2015)[38]	Wagholikar(2012)[39]	Esposito(2011)[40]	Kuo(2011)[12]
1	Was there a clear statement of the aims of the research?	1	1	1	1	1	1	1	1	1	1	1	1	1	1	1	1	1	1	1	1
2	Is a qualitative methodology appropriate?	1	1	1	1	1	1	1	1	1	1	1	1	1	1	1	1	1	1	1	1
3	Was the research design appropriate to address the aims of the research?	1	1	1	1	1	1	1	1	1	1	1	1	1	1	1	1	1	1	1	1
4	Was the recruitment strategy appropriate to the aims of the research?	1	1	1	1	1	1	1	1	1	1	1	1	1	1	1	1	1	1	1	1
5	Was the data collected in a way that addressed the research issue?	1	1	1	1	1	1	1	1	1	1	1	1	1	1	1	1	1	1	1	1

6	Have ethical issues been taken into consideration?	0	0	0	0	1	0	0	1	0	0	0	0	0	0	0	0	0	1	0	0
7	Was the data analysis sufficiently rigorous?	1	1	1	1	0	1	0	1	1	1	0	0	0	1	1	1	1	0	1	1
8	Is there a clear statement of findings?	1	1	1	1	1	1	1	1	1	1	1	1	1	1	1	1	1	1	1	1
**Outcomes of the research**																					
9	Researcher(s) have discussed the contribution of the study to the existing knowledge or understanding:	1	1	1	1	1	1	1	1	1	1	1	1	1	1	1	1	1	1	1	1
9	Researcher(s) have identified new areas where research is necessary:	1	1	1	1	1	1	1	1	1	1	1	1	1	1	1	1	1	1	1	1
9	Paper has addressed whether or how the findings can be transferred to other populations or considered other ways the research may be used:	1	1	1	1	0	0	1	1	1	1	1	1	1	1	1	1	0	1	1	1
	**Quality score**	0.91	0.91	0.91	0.91	0.82	0.82	0.82	1.00	0.91	0.91	0.82	0.82	0.82	0.91	0.91	0.92	0.82	0.91	0.91	0.91
